# Virtual external implementation facilitation: successful methods for remotely engaging groups in quality improvement

**DOI:** 10.1186/s43058-021-00168-z

**Published:** 2021-06-22

**Authors:** Christine W. Hartmann, Ryann L. Engle, Camilla B. Pimentel, Whitney L. Mills, Valerie A. Clark, Virginia C. Keleher, Princess Nash, Corilyn Ott, Therasia Roland, Sharon Sloup, Barbara Frank, Cathie Brady, A. Lynn Snow

**Affiliations:** 1 Center for Healthcare Organization and Implementation Research, VA Bedford Healthcare System, 200 Springs Road (152), Bedford, MA 01730 USA; 2 Department of Public Health, Zuckerberg College of Health Sciences, University of Massachusetts Lowell, Lowell, MA 01854 USA; 3 Center for Healthcare Organization and Implementation Research, VA Boston Healthcare System, Boston, MA 02130 USA; 4 New England Geriatric Research Education and Clinical Center, VA Bedford Healthcare System, Bedford, MA 01730 USA; 5 Department of Population and Quantitative Health Sciences, University of Massachusetts Medical School, Worcester, MA 01605 USA; 6 Center for Innovation in Long-Term Services and Supports, Providence VA Medical Center, Providence, RI 02908 USA; 7 Department of Health Services, Policy and Practice, School of Public Health, Brown University, Providence, RI 02912 USA; 8 Tuscaloosa Veterans Affairs Medical Center, Tuscaloosa, AL 35404 USA; 9 School of Nursing and School of Medicine, University of Alabama at Birmingham, Birmingham, AL 335294 USA; 10 B&F Consulting, 69 Locust Terrace, Warren, RI 02885 USA; 11 Alabama Research Institute on Aging and the Department of Psychology, University of Alabama, Tuscaloosa, AL 35487 USA

**Keywords:** Implementation facilitation, External facilitation, Virtual, Methods, Quality improvement, Remote, Strategies

## Abstract

**Background:**

Relatively little guidance exists on how to use virtual implementation facilitation to successfully implement evidence-based practices and innovations into clinical programs. Yet virtual methods are increasingly common. They have potentially wider reach, emergent public health situations necessitate their use, and restrictions on resources can make them more attractive. We therefore outline a set of principles for virtual external implementation facilitation and a series of recommendations based on extensive experience successfully using virtual external implementation facilitation in a national program.

**Model and recommendations:**

Success in virtual external implementation facilitation may be achieved by facilitators applying three overarching principles: pilot everything, incorporate a model, and prioritize metacognition. Five practical principles also help: plan in advance, communicate in real time, build relationships, engage participants, and construct a virtual room for participants. We present eight concrete suggestions for enacting the practical principles: (1) assign key facilitation roles to facilitation team members to ensure the program runs smoothly; (2) create small cohorts of participants so they can have meaningful interactions; (3) provide clarity and structure for all participant interactions; (4) structure program content to ensure key points are described, reinforced, and practiced; (5) use visuals to supplement audio content; (6) build activities into the agenda that enable participants to immediately apply knowledge at their own sites, separate from the virtual experience; (7) create backup plans whenever possible; and (8) engage all participants in the program.

**Summary:**

These principles represent a novel conceptualization of virtual external implementation facilitation, giving structure to a process that has been, to date, inadequately described. The associated actions are demonstrably useful in supporting the principles and offer teams interested in virtual external implementation facilitation concrete methods by which to ensure success. Our examples stem from experiences in healthcare. But the principles can, in theory, be applied to virtual external implementation facilitation regardless of setting, as they and the associated actions are not setting specific.

**Supplementary Information:**

The online version contains supplementary material available at 10.1186/s43058-021-00168-z.

Contributions to the literature

- Supporting implementation of evidence-based interventions through virtual external facilitation offers opportunities to extend implementation activities’ reach.
- We developed a set of principles for successful virtual external implementation facilitation based on our experience supporting a national quality improvement program.
- Overarching principles are pilot everything, incorporate a model, and prioritize metacognition. Practical principles are plan in advance, communicate in real-time, build relationships, engage participants, and construct a virtual room.
- We give examples of how to enact practical principles to promote success.

## Background

Facilitation is considered a “core ingredient” for successful implementation of evidence-based practices and innovations in complex clinical programs [1]. A facilitation implementation strategy comprises a role (facilitator) and a multi-faceted set of actions (interactive problem-solving and provision of interpersonal support) with individuals, groups, and organizations to enable adoption of innovations into routine practice in the context of a quality improvement (QI) process or implementation study [2]. Facilitation aims to create a supportive environment that enables facilitators and innovation stakeholders to exchange knowledge, identify barriers to implementation, and develop processes to overcome those barriers [3]. Because implementation facilitation relies on continuous relationship- and skill-building, it usually involves a blend of in-person and virtual interactions [4].

External facilitation refers specifically to the role individuals outside an organization have in helping those within the organization implement evidence-based interventions into routine practice. External facilitation has multiple purposes: identifying and resolving problems, communicating, and building and supporting the relationship between the organization and the researchers [5]. Identifying and resolving problems can involve, among other things, identifying barriers, sharing viable solutions and options, helping set realistic goals, and providing networks of peer sites. Communicating may mean providing regular contact and information, monitoring data and possible solutions for problems, and establishing links among sites. And relationship building and support includes providing reassurance, encouragement, empowerment, and mentorship.

Virtual external implementation facilitation is important for implementation studies and programs. Virtual interactions may expand the public health impact of successful interventions [6] by enabling a wider reach than in-person only interactions. Studies conducted during the COVID-19 pandemic, for example, support the potentially greater reach of fully virtual programs [7, 8]. Emergent public health situations may also necessitate transition from in-person to virtual interactions. The COVID-19 pandemic and consequent implementation of social distancing guidelines forced many educational endeavors, laboratories, and non-COVID-19 research efforts either to quickly transition to remote interactions or else temporarily halt their activities [9–11]. Under these conditions, virtual facilitation offered opportunities for teams to remain connected, adapt their plans, and continue their work [12, 13]. In-person facilitation also frequently requires resources (personnel, time) that may be cost-prohibitive. Compared to in-person interactions, virtual team engagement involves fewer travel-related costs, enables more flexible scheduling, may result in greater likelihood of stakeholder attendance and fewer disruptions to clinical responsibilities, and can enhance tracking and archiving of facilitation activities [14]. Taken together, these reasons offer a compelling case for external facilitation implementation to proactively consider how best to leverage advances in teleconferencing and videoconferencing technology.

Our team has extensive experience in virtual external implementation facilitation. In 2017, the Department of Veterans Affairs (VA) Office of Geriatrics and Extended Care created a national QI program for VA’s 134 nursing homes (Community Living Centers, CLCs). The CLCs’ Ongoing National Center for Enhancing Resources and Training (CONCERT) supports CLCs in implementing evidence-based practices for QI [15]. CONCERT, guided by the principles of relational coordination and person-centered care [16–18], aids CLCs in shifting from a quality assurance approach (focusing on remedial actions and retrospective data) to one of high-involvement performance improvement (all staff and managers involved in a prospective, preventative, evidence-based approach to care). Since 2018, CONCERT has held successful 6- to 9-month long learning collaboratives to help CLCs implement evidence-based practices.

We conducted these collaboratives using blended facilitation. Facilitators internal to each site (a 4-person leadership team) worked with external facilitators (members of the CONCERT team). The collaboratives involved interacting with eight CLCs (August 2018 to April 2019; mixture of in-person plus virtual external facilitation), thirty-one CLCs (April 2019 to August 2019; exclusively virtual external facilitation), and ninety-five CLCs (January 2020 to April 2021; in-person facilitation in January 2020 and virtual external facilitation thereafter). External facilitation consisted of facilitating multi-day learning sessions, conducting site-level consultations, conducting individual site as well as group-level coaching, conducting interactive webinars, and communicating with and across sites through phone calls, email, office hours, newsletters, and a web-based site. Internal facilitation was exclusively in-person until the COVID-19 pandemic in March 2020, after which it varied by site but remained mostly in-person.

During the learning intensives, our team used a continual QI process. During multi-day learning sessions and site visits, we met daily to debrief, to learn from that day’s successes and challenges and improve the next. We also met weekly as a team; each meeting included a facilitation update and problem-solving time to harness the power of the team to solve facilitation issues. As of April 2020, we began an additional, weekly, 2-h facilitator development training, in which the team, led by expert consultants, focused exclusively on honing their facilitation skills. In preparation for this paper, we reviewed the extensive written record of these various team meetings and met iteratively as a team to condense what we learned. Based on this, we provide the following recommendations for enabling successful larger-scale virtual implementation facilitation with groups.

## Recommendations

Many principles that are critical to successful virtual implementation are also important in any implementation facilitation effort. External facilitators have traditionally often done some of their work virtually. But because a completely virtual environment means people are never in close physical proximity, in our experience, virtual external implementation facilitation requires special consideration beyond and different from in-person external implementation facilitation. Below, we describe three overarching principles of virtual external implementation facilitation: pilot everything, incorporate a model, and prioritize metacognition. We also describe five practical principles—plan in advance, communicate in real time, build relationships, engage participants, and construct a “room”—and how to enact these.

### Overarching principles

#### Pilot everything

Key to virtual external implementation facilitation success is a continuous QI mindset. Because operating virtually means you are often navigating unfamiliar territory, it is important to start small and use plan-do-study-act cycles to continually refine your processes. To this end, external facilitation team members should take the approach that all applications of the practical principles below are pilot projects that the team studies, discusses, and modifies to try to improve. Holding regular facilitation-team debriefs and trainings are crucial components of this process. To help gain objective feedback, it helps to assign some team members roles as outside observers, where they step outside their team roles during certain portions of the external facilitation experience (such as shadowing on calls or being note-taker during a larger learning session) to observe and provide objective input during the team debrief.

#### Incorporate a model

A model can help guide your virtual external implementation facilitation process by providing clear goals. In our national work, we followed the Institute for Healthcare Improvement’s breakthrough series model [19]. This model emphasizes the importance of the external facilitators in bringing implementation-site participants together in iterative cycles to learn about a focused topic, facilitating these participants’ developing action plans, and supporting their implementing the action plans in their local environments, including helping identify and resolve problems. In our work, this model helped focus the actions we took to enact the practical principles below. The model, for example, reminded us to always emphasize the importance of the participants’ learning from peers as well as recognized experts. And its emphasis on continuous QI as the participants implement the evidence-based practice necessitated our also continually improving our communication and support mechanisms. The breakthrough series model is only one example; external facilitators should incorporate the model that fits their work best.

#### Prioritize metacognition

Adults learn new information best when they quickly have opportunities to apply it and those opportunities are coupled with speaking or writing about what happened when they applied it and with reflecting on what they will do when applying it next [20–22]. This thinking process is called metacognition. By incorporating structured opportunities for both external facilitators and participants to have metacognitive experiences, preferably by talking in groups, information about the new material is encoded into their long-term memories, making them more likely to act on it at their next opportunity [23–25]. This enables both external facilitators and participants to continually improve. An example of how we enacted this principle was by conducting immediate, daily, external facilitator debriefs after any prolonged facilitation efforts with participants (e.g., after a learning session or a site visit).

### Practical principles

#### Plan in advance

Advance planning is critical to the success of any external implementation facilitation endeavor. But virtual situations require additional planning to compensate for the lack of ability to work together quickly onsite to compensate for the unexpected. In an in-person environment, if a piece of technology fails or a presenter shows up late, for example, facilitators can avail themselves of numerous impromptu ways to hold participants’ attention. These scenarios play out differently when people do not share the same physical space. Planning ahead for virtual external implementation facilitation, therefore, requires not only preparing for how you would like things to proceed under ideal conditions but also planning for failure and how to avoid or deal with the many things that can go wrong.

#### Communicate in real time

Communication is key to external facilitation. In a virtual situation, external facilitators are likely not in the same location as the participants. In the case of external facilitation endeavors that involve multiple external facilitators simultaneously, external facilitators may also not be in the same location as each other. In addition, virtual communication may or may not involve video. Communication in these situations, therefore, involves more than would be required in a room where people can talk or gesture to one another. External facilitators must prepare to meet participants at the participants’ virtual communication comfort level. For scheduled interactions, external facilitators should develop a communication mechanism that enables real-time trouble shooting. This may mean keeping an eye on email or messaging platforms when holding a support call, so anyone with technical difficulties can inform facilitators. Many virtual meeting platforms enable behind-the-scenes communication. When there are multiple facilitators for a training, for example, facilitators benefit from practicing with these and other rapid communication mechanisms such as group text messaging to ensure they can inform, trouble shoot, and brainstorm in real time to support participants.

#### Build relationships

A virtual environment increases the danger that relationship-building among participants and between participants and external facilitators may be more difficult or may not happen as quickly or as well as in an in-person environment. Participants, for example, cannot turn to the person next to them at a table; external facilitators cannot walk around the room or—if there is no video or there are multiple people on a video call—read non-verbal cues. For external facilitators, it is also much more difficult to gauge the “temperature” of a room and react accordingly to increase or modify the level or type of support. Virtual external implementation facilitators must consequently be more consistently vigilant about generating opportunities to develop and support relationships among and with participants. This may mean working with participants to pilot different options, finding the best fit for particular circumstances; creating deliberate, regular opportunities for participant feedback; and individualizing and tailoring external facilitation approaches more than might be required under in-person conditions.

#### Engage participants

Participant engagement in a virtual environment also poses particular challenges from an external facilitation perspective, because participants may experience competing demands for their time that they would not experience if interactions with facilitators were in person. Without the peer pressure of a physically communal activity and with the added pressures of one’s local environment and multitasking temptations or requirements, it is also easier for participants to disengage partially or completely. And in a completely virtual environment, facilitators may not be immediately aware of such disengagement when it occurs. Knowing that participants may be distracted at some points, it is critical to include presentation slides that clearly spell out the teaching points at multiple locations in the presentation so that if a participant is distracted for a short period, they will be able to catch up. Building in and enacting specific techniques aimed at fostering participant engagement throughout the virtual external implementation experience—such as more frequent but shorter duration check-ins, participating in regularly scheduled meetings at the participant’s site instead of relying only on separate facilitation meetings, multiplying contact options, or creating brief but meaningful newsletters or important topic webinars—is thus critical to success.

#### Construct a virtual room

Precisely because no actual room exists in a virtual environment, it is critical that participants *feel* a sense of being together in a virtual space with both their peers and the external facilitators. External facilitators must therefore use specific methods to create opportunities for supporting these needs by recognizing and taking advantage of opportunities virtual environments afford, such as using logos and other branding, building togetherness by acknowledging who is participating, and systematically calling on participants so everyone’s voice is heard.

### Enacting practical principles

Enacting the practical principles described above is possible when external facilitators take multiple actions. We describe these below. Table 1 augments these descriptions by using example illustrations from our national work.

**Table 1:** Enacting practical principles: actions, descriptions, and examples

Actions	Descriptions and examples
Assign facilitation roles	There may be several different facilitator roles (e.g., greeter, agenda facilitator, technical support) within a single virtual interaction. Be sure the external facilitators are clear on who is covering which facilitation roles. **Example**: The greeter should welcome participants and avoid awkward silences while participants are gathering (e.g., “Good morning, this is Ryann from the CONCERT team. The program will start in just a few minutes. In the meantime, please type your name and location in the chat box.”). Once the program starts, the greeter hands off to a second team member who facilitates the content of the meeting. While the content facilitator directs the program, another team member troubleshoots individual technical issues and/or manages chat boxes.
Create small cohorts	If there are a large number of participants, deliberately separate participants into smaller groups (organized by facility, location, role, any other relevant characteristic) to enable engagement, sharing, and encourage more meaningful interactions. It may be important to construct smaller groups that avoid hierarchical dilemmas (e.g., separating senior leaders from frontline staff for potentially volatile or personal conversations or mixing sites so supervisors are not in the same group as their own supervisees). **Example**: During the completely virtual external facilitation collaborative the 31 participating CLCs (> 120 individual participants) were separated into 3 cohorts for the duration of the collaborative. During a multi-day learning session cohort 1 (n = 10 CLCs, ~ 40 individual participants) was further divided into 3 smaller breakout groups. During the learning session, the 3 small groups each participated in separate online breakout sessions to discuss performance improvement projects they had implement in their individual CLCs. External facilitators present at each breakout session facilitated discussion across participants, enabling them to highlight their keys to success and other lessons learned. This facilitated discussion was used to help build PowerPoint storyboards for each small group to present during the next segment of the learning session. All small group assignments and connection information for the breakout session were included in the agenda, which was sent to all participants prior to the start of the learning session.
Provide clarity and structure for interactions with/among participants	Explicitly outline for the participants who will be serving in the various facilitation roles. **Example**: The attendance monitor and facilitator contacts no-shows if attendance is mandatory and keeps a database for monitoring the facilitation’s reach. The recording manager starts and stops the recording if the interaction will be recorded. The chat box moderator monitors the chat box or other communication mechanism and informs the facilitator(s) if there are issues or questions that need to be addressed. The presentation moderator shares screens and advances slides. The technical trouble shooter helps team members and participants with technical connection or other issues.
Structure content	Providing structure for the program’s content ensures participants all have similar experiences and focus on the program’s content. **Example**: Create a structured action plan that reinforces learning objectives for participants by guiding them through the plan in a stepwise fashion offline. We provide an example of a templated action plan as an online supplement.
Use visuals	Adding visual components to an audio presentation will enhance participant engagement and keep their attention. Be succinct and use graphics to help convey your message. **Example**: Construct PowerPoint slides with less text and more content-related graphics. If presenting a PowerPoint or other slides, write the important points in the notes section and keep your audience’s attention by having few words on the slides. Before breakout sessions, share a summary slide to remind participants about key issues and guide small group discussion. These bulleted talking points (e.g., please discuss the following: (1) your lessons learned, (2) what you will do differently now that you have learned this new information, (3) how you will take this forward) help participants share their experiences and learn from others.
Build in onsite activities separate from virtual experience	Create time in the agenda for participants to immediately apply learned knowledge in their local setting. Give participants a relatively simple task and reconvene the virtual group to discuss how it went and how to move forward. **Example**: Following a virtual presentation about how to implement a new quality improvement activity, participants from each team are given an hour to practice the activity together locally. Participants then return to the online platform to discuss how the activity went and create action plans for implementing the activity more broadly in their local site.
Create backup plans	Have a plan in place in case there are issues with technology and be sure to have a way to communicate with your team outside of the online platform. **Example**: The external facilitation team connects to the online platform to practice delivering the evidence-based implementation content, with assigned team roles in place. Technical difficulties are identified, so the team decides to create a text message group to use the day of the event. This enables the team members to reach each other about technical difficulties or suggestions in real time during the virtual presentation.
Engage everyone	Facilitators should invite each participant into conversations (via audio or chat), being mindful of group dynamics and continually focusing on collaborative subjects. **Example**: Conduct round robin check-ins during audio sessions in which all participants take turns sharing their experiences. Round robin sharing sessions can then transition to facilitated discussions in which participants and facilitators help each other brainstorm challenges and next steps.

#### Assign key roles

Successful virtual external implementation facilitation requires having clear roles that both facilitation team members and participants recognize and understand. Key among these roles is that of the greeter. A greeter is an identified external facilitation team member who greets everyone who enters a virtual room and who keeps the conversation flowing until the formal agenda begins. The greeter needs to know who will join the meeting (both participants and presenters), who will facilitate the agenda, and the timing of various aspects of the meeting. The greeter should always be the first person to enter the virtual room, so they can identify and interact with participants as they join, welcoming them and assuring them they are in the right place. If possible, the greeter should be on screen or speaking over a placeholder slide with the program name or agenda and start time. The greeter sets the first tone, which should be upbeat and friendly. Greeters should ensure there is no extended silence as everyone waits for the meeting to begin, instead immediately engaging participants. Ahead of time, the greeter should prepare a list of questions to ask, topics about which to chat with participants, or topical information to share. Greeters should also keep the perspective of the newly entering participant in mind. If the greeter is working a virtual room with many participants, every 60 seconds or so, they should clearly identify what they are doing, e.g., “If you are just coming on, welcome. We will begin at the top of the hour. I’m Sharon, and I’m here to welcome you. If you are having any technical difficulties, here are some troubleshooting resources.” If people cannot see each other, when new participants join, the greeter should always identify who is already in the meeting in as much detail as possible, creating a sense of a virtual room. If attendance is expected or mandatory, the greeter should check off participants as they join and, behind the scenes, alert other team members to reach out to those who have not joined the meeting by the start time, to ensure they have the proper information needed to connect or help with technical difficulties.

When the meeting begins, the greeter announces publicly who is in the meeting (or shares some other metric of attendance) and introduces the external facilitator and any internal facilitators (facilitators who are located at the sites). In addition to their main role of engaging with and helping participants implement the intervention, the facilitators introduce topics, announce transitions, and continually orient participants to where the program stands regarding the agenda. They remain flexible, supporting participant input, and delegate troubleshooting tasks to other team members. Because participants’ first and final impressions of any interactions are important, facilitators should prepare and rehearse the main portions of their role, including opening and closing remarks and how they will react if unanticipated challenges arise. Their goal is to engage the participants and remain calm if things do not go according to plan.

External facilitation team members should also take responsibility for other roles to keep the virtual activities flowing smoothly. This works best if team members have opportunities to practice the roles ahead of time. The following is a selection of important roles that apply in various situations; a team member can have more than one role.

1. Attendance monitor and facilitator (contacts no-shows if attendance is mandatory and keeps a database for monitoring the facilitation’s reach)
2. Recording manager (starts and stops the recording if the interaction will be recorded)
3. Chat box moderator (moderates and monitors the chat box or other communication mechanism and informs the facilitator(s) if there are issues or questions that need to be addressed)
4. Presentation moderator (shares screens and advances slides)
5. Technical trouble shooter (helps team members and participants with technical connection or other issues)

Video and audio conference platforms usually enable a private chat feature but may not support a sub-group chat. Consider creating a group chat via cell phone for members of the external and internal facilitation teams. This has the advantage of not being dependent on any one platform’s functionality and enables alerts and problem-solving conversations to occur behind the scenes.

#### Create small cohorts

If the participants number more than a dozen individuals, having meaningful interaction as a large group during the virtual implementation facilitation becomes difficult. Breaking the larger group into smaller facilitated cohorts that meet separately during selected portions of a meeting overcomes this challenge. It enables meaningful sharing among participants and enables them to set their own agenda items more easily. One way to do this is to randomly assign participants behind the scenes. Alternatively, a deliberate, ahead-of-time purposive division into groups based on known relevant characteristics may also increase diversity during meaningful interactions. To promote richer, more inclusive discussions, the external facilitation team can help construct these smaller groups with the participants themselves and/or any internal facilitators.

It is critical that participants have a clear plan and supporting materials for the small groups before separation from a larger group. External facilitators can work with sites to create downloadable or sent-ahead-of-time handouts with exercises, roles, time limits, and troubleshooting information. Consider showing the small group instructions with screen sharing and walking participants through the instructions before sending them to the small groups, no matter how clear your supporting materials may be. And always allow for questions if members get confused. Participants should also have a way to reach an identified facilitation team member(s) in case of trouble. Some platforms do not have clear ways of calling for help. Clarify for participants how they will get help if they get in their breakout groups and they are confused.

Having smaller groups work together can foster a sense of community and collaboration among participants. They can help each other as peers, coaching one another through the application of what they have learned, focusing on each participant’s strengths. Sharing experiences also helps reinforce concepts and techniques.

#### Provide clarity and structure for interactions

The first interaction of participants with a program is often through an initial agenda distributed ahead of time, constructed either by external facilitators or, ideally, by external and internal facilitators and participants working together. This agenda should be clear to a naïve reader. Icons or other visuals can be used to identify repeated actions or clarify the contents of longer agendas that may otherwise overwhelm participants with too much text. In addition to presenting information about content, the agenda should also set expectations for participant interaction and engagement.

As indicated above, facilitators can provide structure to meetings. At the beginning of the meeting, the facilitator can state the mutually understood objectives and support the participants in meeting those objectives throughout. At the end of the meeting, the facilitator can provide closure by recapping what the facilitation team and the participants will each do as next steps. The external facilitator can also provide structure by ensuring the meeting adheres to its schedule. If urgent issues arise during the session, the external facilitator can indicate the facilitation team will follow up, change future sessions, or adjust the remaining agenda items. If changes to the meeting time need to be made, make them during a break for the participants and keep the meeting and meeting section start and end times the same, as participants have likely structured their schedules around these times.

#### Structure content

Structuring as much as is feasible will help ensure core  intervention components are described, reinforced, and implemented. Having structured handouts and notes templates for exercises or other participant experiences help participants understand the evidence-based implementation’s goals, focus their efforts, and share information with fellow participants or others. They give participants tools to capture meaningful data and information and help participants with the hands-on application of knowledge they have gained. To the extent it is desirable, they also ensure all participants have similar experiences. Example structured content includes action plans, report back templates, and train-the-trainer tools. For these, the visuals and graphics should be the same as those used in any program presentations, so participants can easily match program content to the structured content. Ideally, structured exercises and experiences will be created in a collaborative effort between external and internal facilitators, as internal facilitators have the greatest sense of what will work best for participants from their sites.

#### Use visuals

Visuals provide an important supplement to audio interactions. Visuals give participants another way to learn and engage. In advance of a meeting, for example, it can be helpful to send participants documents for review and input. The visuals in these documents should be clear and designed to orient participants. Draw attention to key pieces of information using color, bolding, highlighting, shapes, or call-out boxes. The goal is to create cognitive ease for participants [26]. External facilitators should think about what is needed to convey implementation-relevant content in a clear, engaging, and succinct way and work with internal facilitators and participants to tailor and adapt materials to site needs. Always pilot test visuals with naïve users or internal facilitators before sending to participants or using in a presentation. What seems meaningful to external facilitators who understand the content may not be meaningful to participants for whom the content is new [27, 28].

#### Build in onsite activities separate from virtual experiences

If possible, it is helpful to build time directly into the virtual external facilitation agenda for participants to immediately apply knowledge gained during an interaction. Collaborate with internal facilitators and/or participants to review agendas for potential activity opportunities—look for areas where it is important to reinforce a concept or technique important for the evidence-based practice implementation. In those places, build an activity that participants can do at their own sites. This should be a relatively simple task and easy for participants to do without much logistical effort or pre-planning (although some pre-planning can be expected if participants are notified of the expectation well in advance of the meeting). Incorporate time in the agenda for participants to do the activity at their site and for them to debrief in smaller cohorts. This way they both gain the applied experience and have the metacognitive experience that comes with discussing it with others.

#### Create backup plans

External implementation facilitation involves being nimble. Interactions with sites and participants may go differently than expected. Issues that may arise include challenges with engaging participants, problems connecting to conferencing platform, and key participants being suddenly absent for unexpected reasons. External facilitators can ensure participants will have the best interaction possible and that the interactions meet the intended goal of the activities by creating backup plans in collaboration with internal facilitators or key participants. If participants are silent or disengaged, for example, have an easy-to-answer set of questions ready. If the video platform does not work, make sure everyone knows a backup conference phone number. If an interaction is planned to be especially large, long, or complex, create a checklist [29]. Having backup plans whenever possible will enable the best use of limited virtual interactions with participants.

#### Engage everyone

Participant engagement is critical for successful virtual external implementation facilitation. The external facilitation team should deliberately invite each participant into the conversation. This is why group size is critical and groups where participants number over a dozen should be broken down into smaller cohorts with separate external facilitators. When having a conversation with participants, whether the conversation is in writing (chat box) or by voice, external and/or internal facilitators should acknowledge off topic comments for later discussion and, when necessary, redirect the conversation to ensure opportunities for all to participate. Having an attendee list helps in this regard, particularly for keeping track of who has spoken and being able to call on people by name or group.

During this process, external facilitators need to be mindful of group dynamics and traditional hierarchical structures, which is why collaborating with internal facilitators is so important. Groups may have leaders and supervisors in them as well as other staff, potentially making it more difficult for staff to speak openly. In such cases, it is best to begin with collaborative subjects and redirect any conversation towards collaboration and group strengths. If certain participants are overpowering or monopolizing the conversation, acknowledge their point, if appropriate, and pull in those who are quiet by calling on them directly to contribute their opinions. Ultimately, try to model and create a safe space for all to contribute, to maximize the impact of the external facilitation.

## Conclusion

The overarching and practical principles described here represent a novel conceptualization of virtual external implementation facilitation that gives a practical structure to a process that heretofore in the literature has been inadequately described. The associated actions we describe are demonstrably useful techniques for supporting the principles and offer teams that are interested in virtual external implementation facilitation concrete methods by which to ensure success. Our examples stem from our experiences in using the principles in healthcare. But they can, in principle, be applied to virtual external implementation facilitation, regardless of setting, as the principles and actions are not setting specific. We encourage broad use of the principles and actions to harness the power that virtual methods can bring to external implementation facilitation.

## Supplementary Information

**Additional file 1.**

## Data Availability

Not applicable.

## Abbreviations

CLC: Community Living Center
CONCERT: Community Living Centers’ Ongoing National Center for Enhancing Resources and Training
QI: Quality improvement
VA: Department of Veterans Affairs
