# Supplementary material for: Virtual external implementation facilitation: successful methods for remotely engaging groups in quality improvement
Source: Implement Sci Commun. 2021 Jun 22;2:66. doi: 10.1186/s43058-021-00168-z (PMC8218278; doi:10.1186/s43058-021-00168-z)
Supplement: Supplementary file 1 — Additional file 1. [file 43058_2021_168_MOESM1_ESM.pdf]

CONCERT Learning Intensive 3 – Learning Session 1 (LS1)

Action Planning Session 1 – Watch List Huddles (WLH)

Day 2: 8:00-8:45am

Overall Action Period 1 (Jan 13-Feb 5) Assignment:* *Have WLH up and running in every neighborhood by Learning Session 2 (Feb 6)

Time	Discussion Items	Action Plan: Who will do what when
8:00 – 8:05	Make assignments of roles, (*you will rotate these roles throughout the learning intensive*):<br>1. Facilitator<br>2. Notetaker<br>3. Timekeeper<br>4. Team Spokesperson<br>Review agenda.<br>In a quick go round share ideas you got from mixed tables.	Facilitator:<br>Notetaker:<br>Timekeeper:<br>Team Spokesperson:
8:05 – 8:10	In a go-round:<br>1. Share your lists of residents you're concerned about and why.<br>2. Share your lists of situations that would put someone on the Watch List and add residents accordingly.<br>Together agree on a first draft Watch List. *Remember your nurse managers and neighborhood staff will add to this watch list.*	First Draft Watch List Includes: (use initials)
8:10 – 8:20	How will you introduce/bring WLHs to:<br>1. Nurse Managers (within 3 business days)<br>2. VAMC leadership (Week 1)<br>3. Other Service lines (by Week 2)	Dates/Times to bring WLHs to:<br>Nurse Managers:<br>VAMC Leadership:<br>Service Lines:

Action Planning should be continued and revised as you return to your CLCs and get feedback from staff.

Time	Discussion Items	Action Plan: Who will do what when
	What content will you share?	<u>Agenda/Content/Roles/Etc.</u>
8:20 – 8:30	How will you pilot WLH within the next 5 working days?<br>*Remember your nurse managers and neighborhood staff will help with these decisions.*<br>To do this you need to:<br>- Determine which neighborhood to pilot<br>- Determine the best time and location<br>- Introduce WLH to your staff<br>- Take notes during WLH<br>- Provide coverage so your staff are free to attend	Pilot Neighborhood:<br><br>What do you think is the best day/time and location for WLH pilot:<br><br>How will you get feedback from staff about the best day/time and location for WLH:<br><br>How do you plan to introduce WLH to Staff:<br><br>How do you plan to take notes during WLH:<br><br>How do you plan to provide coverage:

Action Planning should be continued and revised as you return to your CLCs and get feedback from staff.

Time	Discussion Items	Action Plan: Who will do what when
	It is important for the LI Leadership team to attend the huddles. Determine who from the LI Leadership team will attend which huddles.	LI Leadership team huddle assignments:
	By week 2, you need to hold WLHs in all neighborhoods during day tours.	How will you roll out WLH to day tour on all the neighborhoods by the end of Week 2:
	You will need to mentor Nurse Managers, observe and develop their skills.	How will you mentor Nurse Managers in leading WLH:
	You will need to engage all tours in WLH by Feb. 6	What process will you use to roll out WLH to all neighborhoods, all tours by Feb 6:
	You will need to follow up on action items that require action across service lines.	What process will you use to follow up on action items that require action across service lines?

Action Planning should be continued and revised as you return to your CLCs and get feedback from staff.

Time	Discussion Items	Action Plan: Who will do what when
8:30 – 8:35	To oversee this process, the four of you will need to meet weekly. When and where will the four of you meet to oversee this process?<br>*Remember the four of you will likely have impromptu/informal check-ins about WLHs throughout the week. This meeting is to set aside time to formally discuss your progress and next steps.*	<u>LI Leadership Team Weekly Meeting</u><br>When:<br>Where:
8:35 – 8:45	Create your CLC Storyboard (you will have 3 minutes to present this information at the VISN sharing session 8:50 – 10:00)<br>▶ Tell us about the community your VAMC is in<br>▶ Tell us about your CLC:<br>▶ How many Veterans do you serve?<br>▶ What types of care do you provide?<br>▶ How many neighborhoods do you have and what types of care do you provide?<br>▶ Describe the living space Veterans have<br>▶ Each LI team member introduce yourself<br>▶ What is your role?<br>▶ How long have you been in this role?<br>▶ What do you like most about your job?<br>▶ Our biggest strengths are:<br>▶ Our biggest improvement needs are:	Community:<br># of Veterans:<br># of Neighborhoods and types of care:<br>Living space:<br>Strengths:<br>Improvement Needs:
8:45 – 8:50	*Find your VISN meeting – use seating legend*	*VISN meeting starts at 8:50*

Action Planning should be continued and revised as you return to your CLCs and get feedback from staff.

Use this grid for notes during your VISN sharing session

CLC	Good Ideas	Red Flags	Questions

Action Planning should be continued and revised as you return to your CLCs and get feedback from staff.
